# Cloud-Enabled Microscopy and Droplet Microfluidic Platform for Specific Detection of *Escherichia coli* in Water

**DOI:** 10.1371/journal.pone.0086341

**Published:** 2014-01-27

**Authors:** Alexander Golberg, Gregory Linshiz, Ilia Kravets, Nina Stawski, Nathan J. Hillson, Martin L. Yarmush, Robert S. Marks, Tania Konry

**Affiliations:** 1 Centre for Engineering in Medicine, Massachusetts General Hospital, Harvard Medical School, Shriners Burns Institute, Boston, Massachusetts, United States of America; 2 Fuels Synthesis Division, Joint BioEnergy Institute, Emeryville, California, United States of America; 3 Physical BioSciences Division, Lawrence Berkeley National Labs, Berkeley, California, United States of America; 4 DOE Joint Genome Institute, Walnut Creek, California, United States of America; 5 Department of Computer Science, Technion Institute of Technology, Haifa, Israel; 6 Department of Biomedical Engineering, Rutgers University, New Jersey, United States of America; 7 Department of Biotechnology Engineering, The National Institute of Biotechnology in Negev, Ben Gurion University, Beer-Sheva, Israel; 8 School of Materials Science and Engineering, Nanyang Technological University, Singapore; 9 NRF CREATE program for Nanomaterials in Energy and Water Management, Singapore; 10 Department of Pharmaceutical Sciences, School of Pharmacy Bouvé College of Health Sciences, Northeastern University, Boston, Massachusetts, United States of America; University of California, Irvine, United States of America

## Abstract

We report an all-in-one platform – ScanDrop – for the rapid and specific capture, detection, and identification of bacteria in drinking water. The ScanDrop platform integrates droplet microfluidics, a portable imaging system, and cloud-based control software and data storage. The cloud-based control software and data storage enables robotic image acquisition, remote image processing, and rapid data sharing. These features form a “cloud” network for water quality monitoring. We have demonstrated the capability of ScanDrop to perform water quality monitoring via the detection of an indicator coliform bacterium, *Escherichia coli*, in drinking water contaminated with feces. Magnetic beads conjugated with antibodies to *E. coli* antigen were used to selectively capture and isolate specific bacteria from water samples. The bead-captured bacteria were co-encapsulated in pico-liter droplets with fluorescently-labeled anti-*E. coli* antibodies, and imaged with an automated custom designed fluorescence microscope. The entire water quality diagnostic process required 8 hours from sample collection to online-accessible results compared with 2–4 days for other currently available standard detection methods.

## Introduction

Worldwide water-associated infectious diseases are a major cause of morbidity and mortality [1]. It is estimated that 4.0% of global deaths and 5.7% of the global disease burden are caused by waterborne diseases [1]–[4]. Common waterborne diseases include diarrhea (bacterial, viral and parasitic), schistosomiasis, trachoma, ascariasis, and trichuriasis [1]–[4]. Low income countries are particularly vulnerable to waterborne diseases because of their under-developed infrastructure and poor water management [5]–[14]. Water and sewage distribution systems in high income societies also require pollutant and microorganism monitoring [15].

*Escherichia coli*, found in mammalian feces [16], has been a biological indicator for water quality since the 19^th^ century [16]. Testing for the presence of *E. coli* is obligatory for current water management systems [17]–[19]. Herein, we report a comprehensive system – ScanDrop – for the rapid and specific identification of *E. coli* in drinking water.

The identification of bacteria in a water sample includes two major steps: 1) the capture of target bacteria from the water sample, and 2) the identification of the captured bacteria. Traditional methods for *E. coli* detection include culture, fermentation, enzyme-linked immunosorbent (ELISA), and polymerase chain reaction (PCR) assays [20], [21]. These traditional methods have disadvantages including long identification times (2–4 days), and/or high labor and reagent costs [20], [21]. Despite high costs, rapid tests are necessary to enable quick responses to putative contamination threats. Recently, novel sensors and assays for rapid pathogen detection have been developed, including the capture of whole pathogen cells or molecular fragments for further amplification and identification [22]–[27], with detection methods utilizing a variety of transducing technologies (optical, electrochemical, surface plasmon resonance and piezoelectric) [27]–[40]. Many of these newer methods remain expensive and/or require sophisticated instrumentation, and most have yet to reach the market place. Therefore, there remains a need for alternative platforms for the detection of bacteria in water samples.

It remains challenging to inexpensively perform water quality control testing at multiple locations along a distribution system, and to rapidly process and share the test results. To address these challenges, we have developed the ScanDrop platform. ScanDrop is a self-contained detection platform that enables the online control of water testing at multiple locations along the distribution system. ScanDrop integrates live-bacteria capturing and detection, droplet microfluidics, automated fluorescence microscopy, and cloud-based data management and sharing. Droplet microfluidics, applied in ScanDrop, is an emerging application of microelectromechanical systems (MEMS) technology, where assay reagents and biological sample are confined to the pico-liter reactors, composed of water in oil emulsion [41]–[43]. Small volumes, rapid reagent mixing and non-complex droplet control make droplet microfluidics an attractive choice for the next-generation of high-throughput assays [41]–[43] and herein detection of bacteria in water samples.

In this work, we demonstrate ScanDrop's capability to detect live *E. coli* in water samples. Magnetic beads, conjugated with specific antibodies, were used to quickly and effectively capture *E. coli* from contaminated water. The captured bacteria were then encapsulated into pico-liter droplets containing fluorescently labeled antibodies, for subsequent detection using a proprietary automated optical fluorescence signal registration system. Imaging system control was facilitated by leveraging a cloud-based laboratory automation system, coined Programing a Robot, PR-PR [44]. We envision that multiple ScanDrop systems could be dispatched at multiple locations to form a cloud-enabled water quality assessment network. Each system could be managed in real-time from a remote control center. Such a network could potentially reduce the infrastructure, management, and labor costs required to perform multiple sample analysis and rapidly share results.

## Results and Discussion

### Bead-based *E. coli* capture and detection assay

**Figure 1:**
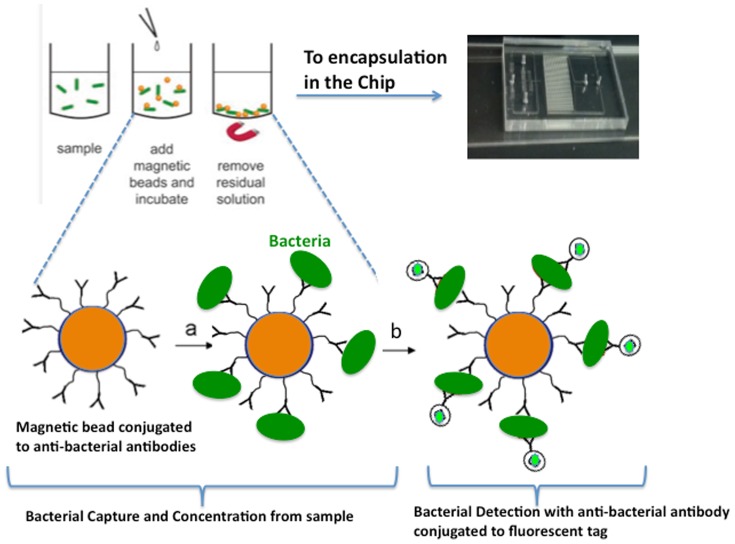
Bacteria capturing and detection assay. Magnetic bead capture of *E. coli* from enriched water samples, and downstream chip encapsulation for fluorescent labeling and detection. 1L of water is passed through a 0.22 µm filter, which is then incubated for 6 hr in LB media. Dynabeads® MAX anti-*E. coli* O157 are added to the resulting cell culture (“sample”), incubated for 20 min, and concentrated via magnet. The beads (potentially conjugated with bacteria) are then co-encapsulated with secondary fluorescently labeled anti-*E. coli* antibodies in the chip and incubated up to 1 hour before imaging.

Herein the isolation of bacteria and detection are conducted utilizing simple magnetic bead based immunoassay thus no bacteria agar plate cultivation step is necessary to identify a presumptive positive sample. This approach saves considerable time and resources. In our approach, magnetic beads conjugated with anti-*E. coli* antibodies are added to a water sample (**Fig. 1**). Within 10 min, the magnetic beads have captured the bacteria (if any) from the water sample. The beads are then concentrated with a simple magnet (**Fig. 1**), and a single immunoassay step labels the captured bacteria with a fluorescent antibody for subsequent detection (**Fig. 1**). Detection protocols are integrated into a droplet microfluidic device to reduce reagent volume and enhance reaction rates.

### ScanDrop Sensor

The ScanDrop sensor consists of two major components: 1) a droplet microfluidic device for bacteria labeling, and 2) a portable fluorescent optical system for signal detection and sharing.

#### Droplet microfluidic device

**Figure 2:**
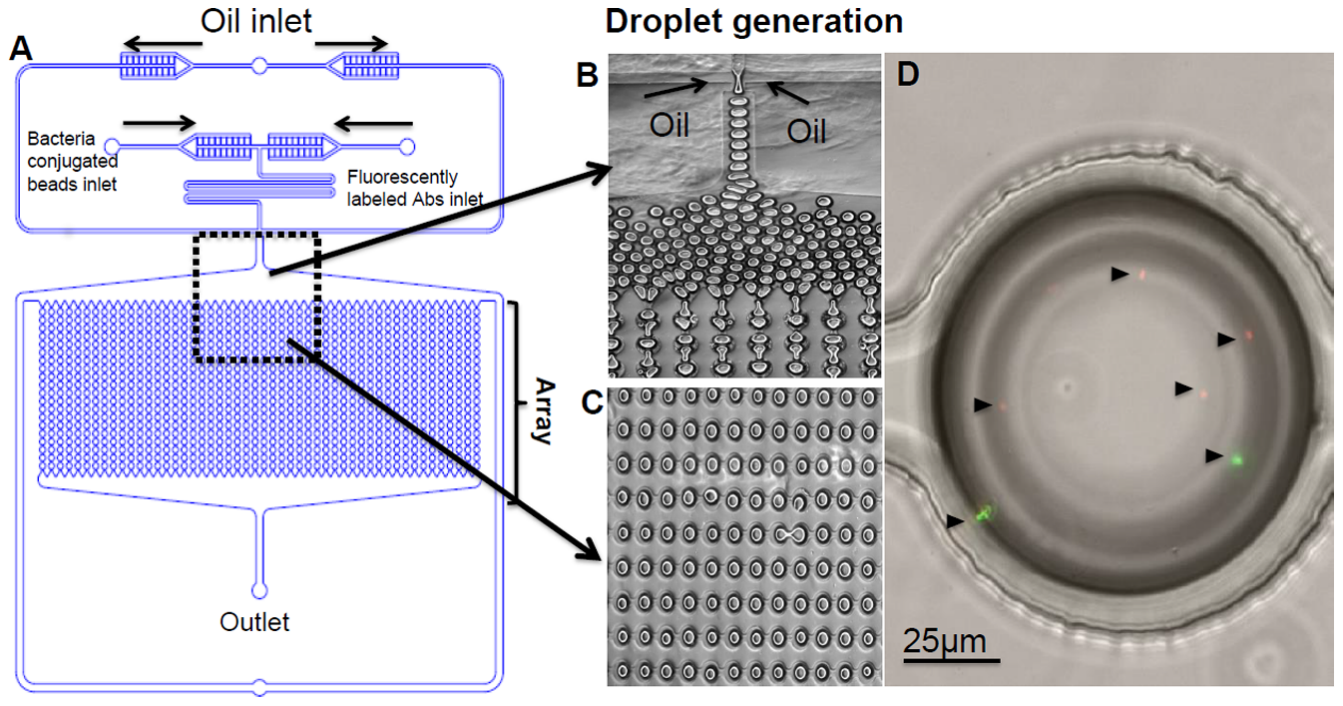
Droplet microfluidic device for bacteria monitoring. **A**) Schematic representation of ScanDrop droplet microfluidic chip and fluid control system. **B**) Droplet generator. **C**) Droplet incubation array (up to 10^3^ droplets can be incubated simultaneously). **D**) Co-encapsulation of GFP- and RFP-expressing *E. coli* inside a single droplet (20× magnification, Zeiss microscopic imaging). Arrows indicate single bacteria cells.

To reduce reagent volumes and detection times, we designed a pico-liter droplet microfluidic chip. The design of poly(dimethylsiloxane) (PDMS) microfluidic device is shown in **Fig. 2A**, the generation of monodisperse droplets in a micro-channel through shearing flow at a flow-focusing zone in **Fig. 2B**, and the resulting droplet array in **Fig. 2C**. Three perpendicular inlet channels form a nozzle, (**Fig. 2A** **rectangle**), independent syringe pumps controlling flow rates for the oil, beads, and fluorescently labeled secondary antibodies streams. Each droplet in the array co-encapsulates fluorescently labeled anti-*E. coli* antibodies with captured bacteria (if any), to generate a localized fluorescent signal for subsequent detection. The chip enables the generation and incubation of 10^3^ droplets with ∼100 micron diameter (∼520 pL). The advantages of this droplet-based array technique include the physical and chemical isolation of beads in droplets, and the rapid and efficient mixing of the reagents that occurs inside droplets providing fast reaction rates [45]–[48]. Importantly, this nano-liter microenvironment also enables gas exchange for bacterial viability if further studies are required [45]–[48]. Previous works in the field of droplet microfluidics showed that the chance to find a cell or a bead inside droplet follows Poisson distribution [49], [50]. This puts certain theoretical limitations of the limit of detection of bacteria of droplet microfluidic system. Clausell-Tormos et al. showed that decreasing the number of cells in aqueous solution that is converted to droplets to less than 10^6^ cell/mL reduced the probability to find droplets with encapsulated cells and increased the number of empty droplets [50]. Therefore, at the end of incubation time we need to get at least 10^6^ CFU/mL of bacteria. The relation between the bacteria in the analyzed sample and the bacteria subjected to encapsulation after enrichment is as follows:
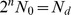
Where N_o_ (CFU/mL) is the initial load of bacteria, n is the number of generations in enrichment phase, N_d_ (CFU/mL) is the concentration of bacteria subjected to encapsulation after enrichment.

Given the generation time of 20 min for *E. coli* in the optimum cultivation condition, to get 10^6^ CFU/mL at the end of 6 hours of incubation (18 generations), proposed in our assay, the initial concentration should be at least 3.5 CFU/mL.

To demonstrate the feasibility of our ScanDrop system for multiplex analysis, we co-encapsulated red florescent protein (RFP) and green florescent protein (GFP) expressing *E. coli* in the same droplet (**Fig. 2D**). Capturing on a bead and latter encapsulation for detection of two different bacteria in the same microenvironment will enable multiplex future studies using several types of beads conjugated with different antibodies that bind different target bacteria and different fluorescent tags. The probability of capturing two different objects in a single droplets were analyzed in [49].

### Optical system

**Figure 3:**
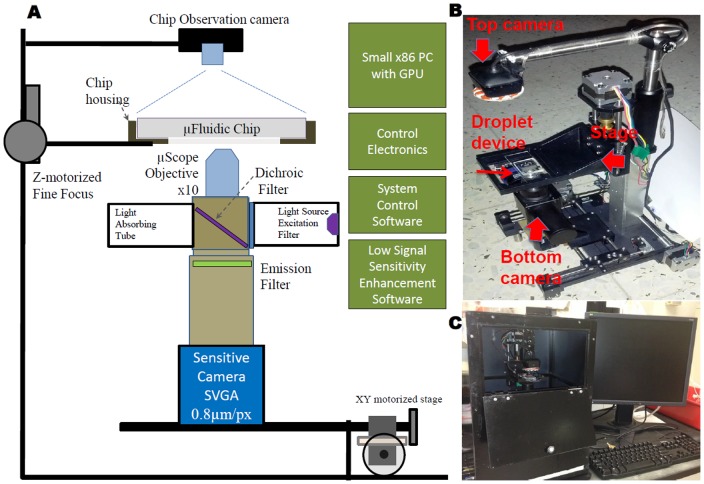
Portable fluorescence microscope system. **A**) Microscope design scheme. **B**) Robotic stage and two cameras (arrows) for chip observation and data acquisition. **C**) ScanDrop system set up, including 2 pressure pumps to create droplets, a microscope imaging system, internet access, and a monitor for local data viewing.

The schematic for the portable optical system for fluorescent signal detection in the droplet microfluidic device is presented in **Fig. 3A**. The system enables remote microscope control as well as simultaneous top and inverted image registration (**Fig. 3B**). The top camera allows for whole chip bright field imaging, while the bottom camera allows for fluorescence imaging with 10× magnification. This combination allows for high-throughput droplet imaging. A robotic stage is used to scan the array of multiple droplets, with an XY microscope scanning range of 45 mm×45 mm and a resolution of 5 µm/step (10 mm/sec). Z-axis focus capabilities include 15 mm travel with 1 µm/step, at 2 mm/sec. The ScanDrop optical system is controlled by Python scripts which can be automatically generated by PR-PR.

### PR-PR cloud-based Laboratory Automation System, and Data Management and Sharing

In this work, we have further developed PR-PR, a biology-friendly high-level language for laboratory automation [44], to control ScanDrop's automated microscopy system and enable ScanDrop to be promptly and easily adjusted to changes in experimental protocol. In PR-PR, transfer of a material (*e.g.*, a liquid) or system component (*e.g.*, a robotic arm) is described by a **S**ource, **D**estination, **Q**uantity, and **M**ethod. For ScanDrop, the **S**ource is the initial coordinates of the microscope stage (XY) and lens (Z), the **D**estination is the final target coordinates of interest, the **Q**uantity is the number of pictures that should be taken, and the **M**ethod specifies imaging parameters: light, filters, and delay between image capture. PR-PR inputs a script for ScanDrop automated microscope control (such as that presented in **Fig. S2**) and outputs a Python script that can directly operate the ScanDrop automated microscope system. The PR-PR script protocol and the resulting data for each experiment are stored in a local folder within the ScanDrop sensor and can shared between users via Dropbox.

### Detection of RFP-expressing *E. coli* in drinking water

**Figure 4:**
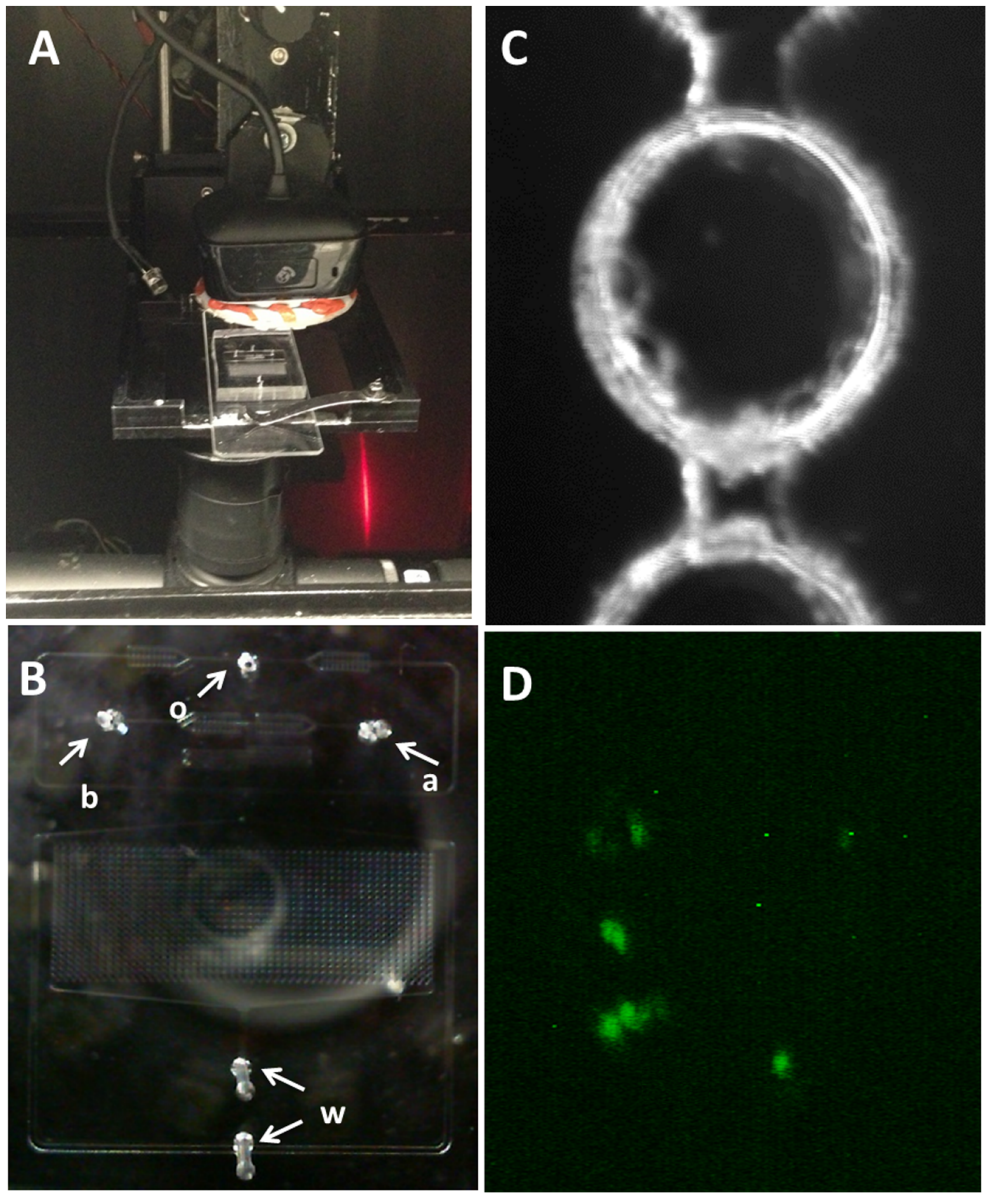
ScanDrop: *E. coli* detection. **A**) Microfluidic chip operating inside the imaging system. **B**) Droplet array as viewed from the top camera. Arrows indicate tubing/chip connection locations as follows: o- oil (inlet); b- beads conjugated with *E. coli* (inlet); a- fluorescently labeled antibody (inlet); w- waste (outlets). **C**) Droplet image as seen from the top camera with white LED illumination. **D**) Antibody green fluorescence indicates the presence of RFP-expressing *E. coli* (positive control).

As a positive control, we tested ScanDrop for the detection of 150 CFU/mL of RFP-expressing *E. coli* in drinking water (**Fig. 4**). The overall assay for *E. coli* detection is divided into three steps: enrichment, capture, and detection. For enrichment, 1L of contaminated water sample was filtered and the filter with captured bacteria was incubated for 6 hours in the LB medium. For capturing, the enriched solution was mixed with Dynabeads® MAX anti-*E. coli* O157 for 10 min and separated by magnet.

**Figure 5:**
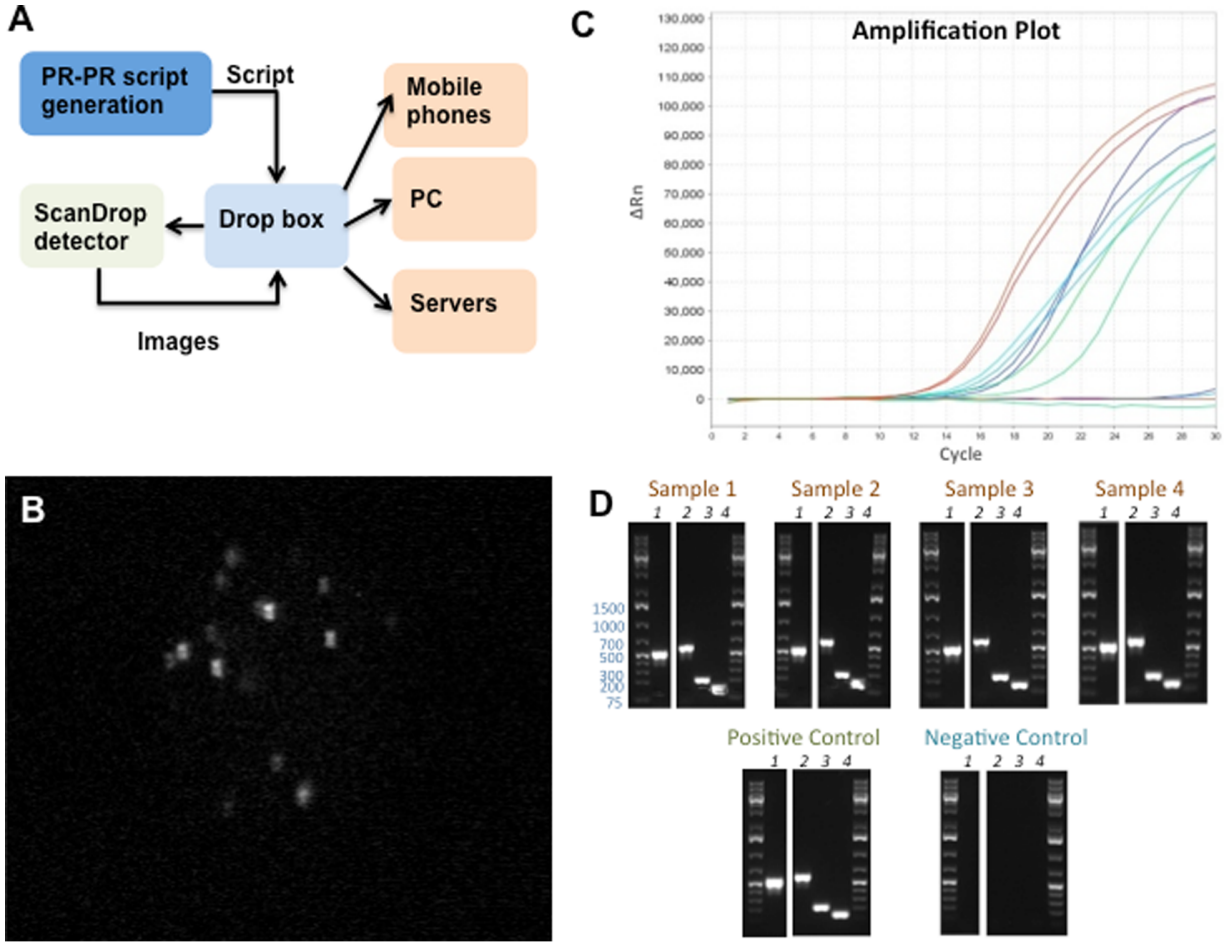
Detection of fecal *E. coli* in drinking water. **A**) ScanDrop detection network. PR-PR generates a Python script to control the ScanDrop detector. The Python script is uploaded to Dropbox, and then run on the ScanDrop detector. The captured images are uploaded to Dropbox, and then distributed to various devices. **B**) Representative ScanDrop image demonstrating fecal *E. coli* detection in drinking water. **C**) Real-time PCR amplification plot for contaminated sample 3, and positive and negative controls. Red curves indicate amplification of primary *16S rRNA* locus, cyan amplification of secondary *16S rRNA* locus, green *tuf* locus, and blue *uidA* locus. Sample 3 and the positive control amplified similarly. The negative control did not amplify. **D**) Gel electrophoresis analysis of PCR reaction products for the four contaminated samples, along with positive and negative controls. For all samples, gel lanes correspond to the amplification of loci as follows: lane 1 - *16S rRNA* primary locus, lane 2 - *16S rRNA* secondary locus, lane 3 - *tuf*, lane 4 – *uidA*.

**Fig. 4B** shows the droplet-based microfluidic chip used to perform the immunoassay described in **Fig. 1**. For the detection step, beads conjugated to bacteria captured from contaminated water sample were co-encapsulated with secondary FITC fluorescently labeled anti-*E. coli* antibodies in the droplet array and incubated up to 1 hr in the chip array at room temperature. **Fig. 4C** shows a representative droplet, and **Fig. 4D** shows the green florescence signal detected in a single droplet containing *E. coli* capture on the bead and tagged by secondary FITC labeled antibodies. The presence of RFP expressing *E. coli* in water was confirmed by PCR (**Fig. 5 C, D**).

### Detection of fecal *E. coli* in drinking water

Next, we tested the ScanDrop system for the detection of fecal *E. coli* in drinking water. We contaminated the water with rat feces and applied the droplet detection assay described above. **Fig. 5A** presents the procedure flow for the detection of fecal *E. coli* in water. **Fig. 5B** shows a representative resulting image, with fluorescence signal indicating *E. coli* contamination. We confirmed the ScanDrop detection with PCR (**Fig. 5 C,D**), which clearly showed that the water samples were contaminated with *E. coli*. The results from the ScanDrop tests were uploaded to Dropbox cloud data storage.

The work presented here demonstrates the potential of automated microscale systems for water quality analysis. To detect *E. coli* in water samples, we developed and demonstrated a bead-based immuno-assay performed with a droplet microfluidic device to reduce reagent volume and enhance reaction rates. We integrated the microfluidic assay with a portable imaging system and remote control automation software. We demonstrated ScanDrop system capabilities through the detection of model coliform bacteria, *E. coli*, in feces–contaminated drinking water. Our successful multiplex detection assay results suggest that simultaneous multiple bacteria detection, using several types of beads conjugated with different antibodies that bind different target bacteria, will be possible with further development. The ScanDrop platform decreased reagent volumes, (the full chip uses 520 nL of reagents, while conventional assay require at least 10 µL of reagents) and allows for results within 8 hours from the time of water sampling. Our results demonstrate that a combination of droplet microfluidics with low cost optics and cloud network can provide a flexible and efficient alternative for pathogen detection in drinking water. The ScanDrop platform has the potential to significantly improve water diagnostics, particularly in low income countries where the infrastructure does not yet exist [51], [52].

## Conclusions

**Figure 6:**
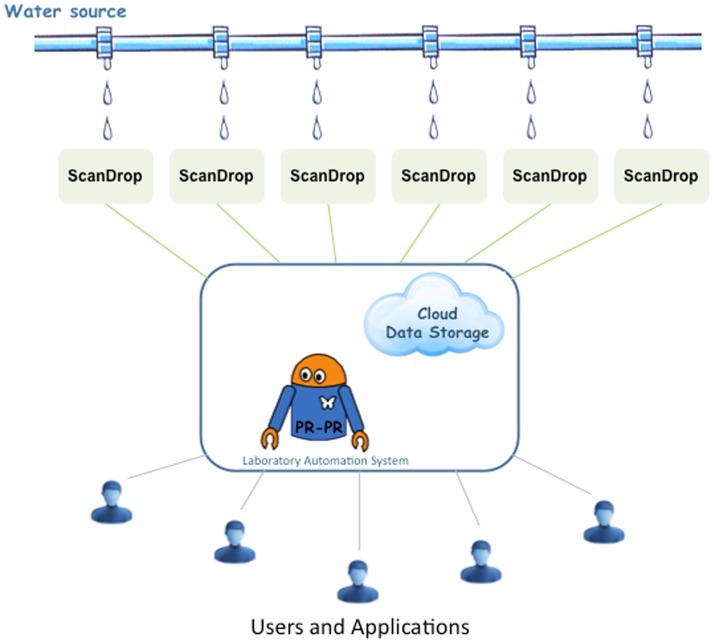
Schematic overview of a ScanDrop cloud-based water quality assessment system. The ScanDrop detector network is enabled by PR-PR and cloud-based data storage. Users send requests for water quality assessment at different locations in the distribution system. ScanDrop detectors perform the tests, the results are stored in the cloud, and the collected data is shared between users and applications.

We developed the ScanDrop platform for *E. coli* detection in water. The platform uses magnetic beads to capture bacteria, droplet microfluidics to encapsulate the captured bacteria with fluorescent antibodies, low cost portable optics for signal detection, PR-PR to facilitate microscopy control and data acquisition, and cloud-based storage for results sharing. The use of droplet microfluidics increases reaction kinetics and reduces reagent volumes (lowering the cost per test), the developed florescence microscopy system allows for data generation in multiple locations, and PR-PR facilitates ScanDrop control. A schematic illustration of an envisioned ScanDrop network for water quality analysis is shown in **Fig. 6**. The ScanDrop network would consist of 1) the PR-PR laboratory automation system and cloud-based data storage for remote control, image capturing, and result sharing; and 2) ScanDrop sensor stations deployed at multiple water distribution locations. The control station would perform image analysis for multiple sensors and shares the test results in the real time with multiple end users. This ScanDrop network could contribute to more rapid, cost-effective, and continuous water quality monitoring systems, with centralized facilities simultaneously monitoring multiple water sampling sites without complex imaging or data processing infrastructure.

## Materials and Methods

### ScanDrop optics system

A custom made, motorized, dual view, computerized portable microscopy system was designed for droplet microfluidic imaging (R&D Engineering Solutions, Netania, Israel). The dual view system was used for the simultaneous imaging of the whole chip (top view camera) and specific droplets (bottom view camera). The top view camera includes: 1280×768 resolution, color sensor, auto/computer-controlled focus, manually configurable [83×50 mm - 30×18 mm] field of view, 640×480 region of interest (ROI), and zoom functionality. The bottom view (microscope) camera includes: 752×582 resolution, monochrome 8.6 µm×8.3 µm pixels sensor, and a 10× objective. A single 3W 468 nm light emitting diode (LED) was used for florescence excitation. A 41017 - Endow GFP/EGFP bandpass fluorescence filter set (Chroma Inc., VT) was used for florescence detection. Top illumination was made by a single 30 mW white LED for chip observation and microscope camera positioning. An embedded ×86 dual core computer with HDMI display port outputs (CompuLab, Israel) was used for the local control of the system. An embedded computer runs custom software, which allows full control of the microscope, including XY position, focus, illumination, image acquisition and enhancement. A system can be controlled manually by the human operator via a standard PC console (keyboard, mouse and monitor). Alternatively, a system can be controlled programmatically via a program written in Python. We further improved the programmatic control aspect of our system by leveraging PR-PR [44], whereby a PR-PR microscope control script (**Fig. S2**) is translated into a Python script that can control the microscope system, as described above. Python script deployment and image retrieval across distributed microscope systems was performed with the Dropbox cloud-based storage service.

### Bacterial strains and plasmids

Plasmids pFAB_SchPMK36GFP and pFAB_SchPMK36RFP (unpublished results, Vivek Mutalik, Drew Endy, and Adam Arkin; see **Fig. S1**), both carrying a kanamycin resistance marker, were transformed into *E. coli* BW25113. These bacterial strains and plasmids, along with their associated information (*e.g.*, annotated Genbank-format DNA sequence files), have been deposited in the public instance of the JBEI Registry [53] (https://public-registry.jbei.org; corresponding Part IDs JPUB_001327-001329). For transformation, 1 µL pFAB_SchPMK36GFP or pFAB_SchPMK36RFP was mixed on ice with 40 µL chemically competent *E. coli* BW25113. The mixture was incubated on ice for 20 min, then placed at 42°C for 45 s (heat shock), and then returned to ice. 200 µL SOC media was then added to each tube of transformed cells and incubated with agitation at 37°C for 30 min. 100 µL of each transformation mixture was plated on solid LB media (Sigma-Aldrich, MO) supplemented with 30 µg/mL kanamycin (Sigma-Aldrich, MO) and then cultured at 37°C.

### Microfluidic device for droplet generation

The droplet microfluidic flow focusing device mask was fabricated by soft lithography. Negative photo resist SU-8 2100 (MicroChem, Newton, MA) was deposited onto clean silicon wafers to a thickness of 150 µm, and patterned by exposure to UV light through a transparency photomask (CAD/Art Services, Bandon, OR). To manufacture consumable devices, Sylgard 184 poly(dimethylsiloxane) (PDMS) (Dow Corning, Midland, MI) was mixed with cross-linker (ratio 10∶1) and poured onto the photoresist pattern, degassed thoroughly and cured for 12 hours at 75°C. After curing, the PDMS devices were peeled off the wafer and bonded to glass slides after oxygen-plasma activation of both surfaces. The microfluidic device was composed of two parts: 1) a droplet forming nozzle (channel cross section 6.25·10^−8^ m^2^) and 2) a 10^3^ droplets storage array (channel cross section 3.13·10^−7^ m^2^). The bonded microfluidic channels were treated with Pico-Sur™ 2 (Dolomite Microfluidics, UK) by filling the channels with 10 µL of the solution as received and then flushing with air. This treatment was done to improve the wetting of the channels with mineral oil in the presence (1% w/w) of the surfactant (span80). 1 mL syringes were used to load the fluids into the devices through Tygon Micro Bore PVC Tubing 100f, 0.010″ ID, 0.030″ OD, 0.010″ Wall (Small Parts Inc, FL). Individual syringe pumps (Harvard Apparatus, USA) were used to control the flow rates of oil and other reagents. To form droplets, the flow-rate-ratio of water-to-oil was adjusted to *Q*_w_/*Q*_o_ = 1.

### Droplet microfluidics multiplex detection assay

*E. coli* expressing GFP or RFP were incubated for 12 hours at 37°C in LB media (Sigma-Aldrich, MO) to 10^6^ CFU/mL and encapsulated into droplets. Fluorescence images were captured on a Zeiss 200 Axiovert microscope using an AxioCAM MRm digital camera and AxioVision 4.8 software at 20× magnification. Each experiment consisted of 4 repeats.

### ScanDrop detection of *E. coli* in water

1 L drinking water was spiked with RFP-expressing *E. coli* to 150 CFU/mL. The spiked water was filtered through a 0.22 µm filter (Corning Inc., NY), and the filter was then inoculated in 10 mL LB media (Sigma-Aldrich, MO) and incubated for 6 hr at 37°C. 20 µL of Dynabeads® MAX anti-*E. coli* O157 beads (Life Technologies, CA) were added to 1.5 mL of the incubation media and further incubated for 20 min on a rotating stage at room temperature (RT). Beads with captured bacteria were separated by magnet and resuspended in 400 µL of Phosphate Buffered Saline (PBS). The resuspended solution was co-encapsulated 500∶1 with green fluorescently labeled anti-*E. coli* antibody (FITC, ab30522, Abcam, MA) in droplet reactors inside the chip positioned on the ScanDrop robotic stage. After a further 1 hr of incubation at RT, images were taken from different locations on the chip. The objective position movements were controlled via PR-PR, and the generated images were automatically uploaded to Dropbox. Each experiment consisted of 4 repeats.

### Drinking water contaminated with rat feces

Fresh feces were collected from rat cages in the animal facility of Massachusetts General Hospital. 1.5 g feces was mechanicaly homogenized in 1 L of drinking water. The contaminated water was filtered twice through a 40 µm filter (BD Falcon™, BD Biosciences, CA). The detection of E.coli in permeate was done by ScanDrop assay (as described in the previous section) and by Real-Time PCR (as described in the following section). Each experiment consisted of 4 repeats.Detection of RFP expressing and fecal origin E. coli by PCR. We chose four primer sets (**Table 1**) to detect E. coli in the prepared drinking water contaminated with rat feces. The primers targeted specific sequences from different loci in the E. coli genome: two primer sets for 16S rRNA, one for *tuf*, and one for *uidA* [54]–[57]. For each primer set, we tested four contaminated samples and a positive and a negative control. Negative controls contained water only, and positive controls contained water supplemented with *E. coli* BW25113. After enrichment of the microbial population (described above), 5 µL of enriched culture was added to 45 µL H_2_O. All samples were incubated 15 min at 98°C and then diluted in additional 100 µL H_2_O. Each 30 µL PCR reaction contained 10 µL of the diluted cell lysate (as template), 10 µL of 3× qPCR master mix (H_2_O 3.3 µL, 5× Phusion HF 6 µL, dNTP 100 mM 0.25 µL, Phusion DNA Polymerase (NEB) 0.3 µL, SYBR® Green II 200× (Molecular Probes) 0.15 µL), and a pair of primers at 5 pmol each. PCR reactions were subjected to thermal cycling (3 min at 95°C, and then 30 cycles of 30 s at 95°C, 30 s at 58°C, and 30 s at 72°C, with a final hold step at 10°C) in a StepOnePlus™ Real-Time PCR System (Life Technologies, CA). We tracked the amplification curves and stopped the PCR amplifications after most reactions plateaued (**Fig. 5c**). We analyzed PCR fragments using electrophoresis by running the PCR products in 1% agarose gels (**Fig. 5d**). Each experiment consisted of 4 repeats.

**Table 1:** Primers and loci used for PCR detection of *E. coli*.

Locus	Primers	T_m_	Amplicon Length
16S rRNA	ECA75F - GGAAGAAGCTTGCTTCTTTGCTGAC	60°C	544 bp
(primary)	ECR619R- AGCCCGGGGATTTCACATCTGACTTA		
16S rRNA	16E1 - GGGAGTAAAGTTAATACCTTTGCTC	60°C	583 bp
(secondary)	16E2 - TTCCCGAAGGCACATTCT		
*tuf* gene	TEcol553 - TGGGAAGCGAAAATCCTG	58°C	258 bp
	TEcol754 - CAGTACAGGTAGACTTCTG		
*UidA* gene	UAL - TGGTAATTACCGACGAAAACGGC	62°C	147 bp
	UAR - ACGCGTGGTTACAGTCTTGCG		

### PR-PR software availability

PR-PR is open-source software under the BSD license and is freely available from GitHub (https://github.com/jbei/prpr), and is also available through its web interface on the public PR-PR webserver (http://prpr.jbei.org).

## Supporting Information

Figure S1 **Plasmid maps for pFAB_SchPMK36GFP and pFAB_SchPMK36RFP.**
(TIF)

Figure S2 **Representative PR-PR script for ScanDrop.** LOCATION declarations define microscope stage (XY) and lens (Z) locations. TRANSFER commands specify the starting and destination locations, the number of pictures to capture, and the capture parameters. In a single TRANSFER statement, multiple sequential destinations can be defined by location offset and number of repetitions.
(TIF)
