# Supplementary figures and images for: Cloud-Enabled Microscopy and Droplet Microfluidic Platform for Specific Detection of Escherichia coli in Water
Source: PLoS One. 2014 Jan 27;9(1):e86341. doi: 10.1371/journal.pone.0086341 (PMC3903517; doi:10.1371/journal.pone.0086341)

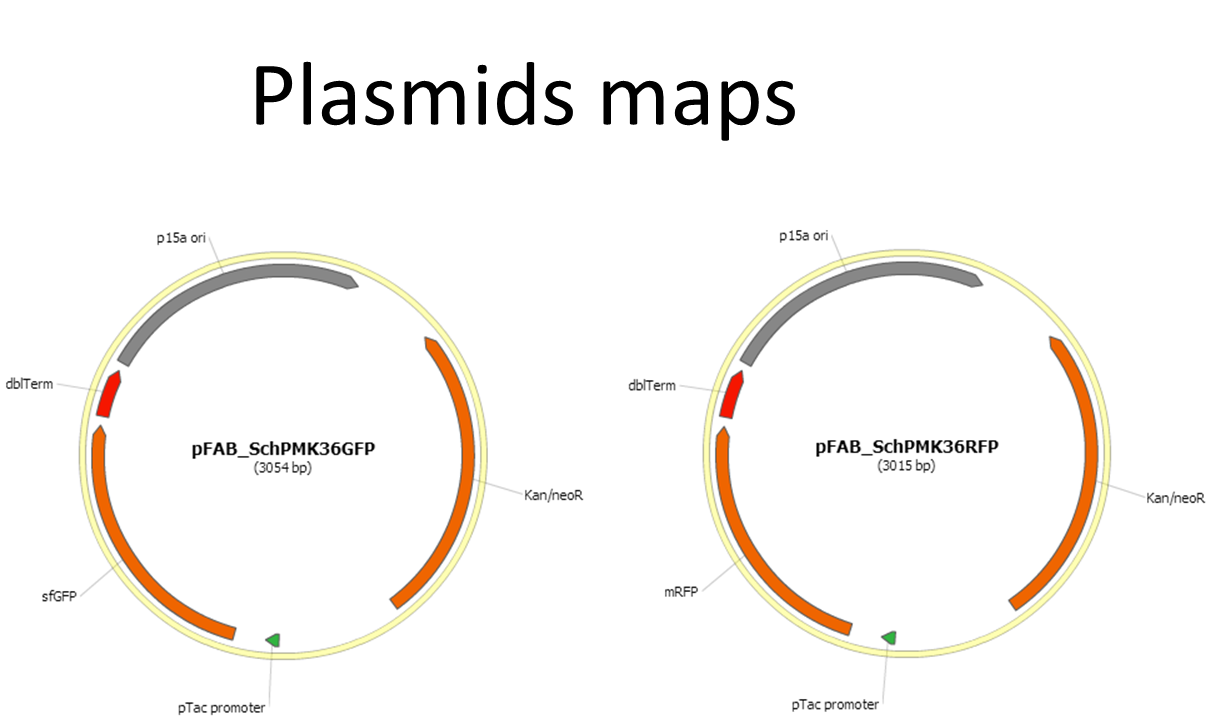

Supplement: Figure S1 — Plasmid maps for pFAB_SchPMK36GFP and pFAB_SchPMK36RFP. (TIF) [file pone.0086341.s001.tif]

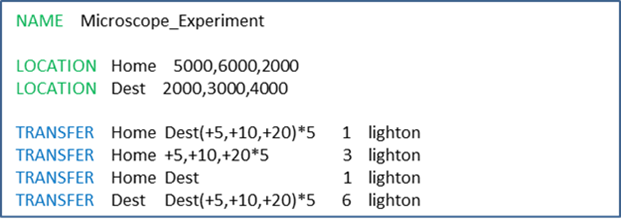

Supplement: Figure S2 — Representative PR-PR script for ScanDrop. LOCATION declarations define microscope stage (XY) and lens (Z) locations. TRANSFER commands specify the starting and destination locations, the number of pictures to capture, and the capture parameters. In a single TRANSFER statement, multiple sequential destinations can be defined by location offset and number of repetitions. (TIF) [file pone.0086341.s002.tif]
